# Influence of Increased Joint Line Obliquity on Survivorship After Lateral Closing-Wedge High Tibial Osteotomy

**DOI:** 10.1177/03635465241270292

**Published:** 2024-08-21

**Authors:** Tianshun Xie, Astrid J. de Vries, Hugo C. van der Veen, Reinoud W. Brouwer

**Affiliations:** † Department of Orthopaedic Surgery, University of Groningen, University Medical Center Groningen, Groningen, The Netherlands; ‡ Department of Orthopaedic Surgery, Martini Hospital, Groningen, The Netherlands; Investigation performed at Martini Hospital, Groningen, The Netherlands

**Keywords:** knee joint line obliquity, survival, risk factors, lateral closing-wedge high tibial osteotomy

## Abstract

**Background:**

Although high tibial osteotomy (HTO) has emerged as a powerful intervention for treating symptomatic medial osteoarthritis and varus malalignment, it can result in an increase in knee joint line obliquity (KJLO) in the frontal plane. Limited current evidence hinders understanding of the effect of increased KJLO on HTO survivorship.

**Purpose:**

To investigate the influence of KJLO and other potential risk factors on the survivorship of lateral closing-wedge HTO.

**Study Design:**

Cohort study; Level of evidence, 3.

**Methods:**

Patients with symptomatic medial knee osteoarthritis and varus malalignment treated with lateral closing-wedge HTO at a single hospital were screened with a minimum follow-up of 5 years. HTO survival rate was assessed using Kaplan-Meier survival analysis. The influence of postoperative increased KJLO (medial proximal tibial angle ≥95°), age (≥55 years), sex (female), preoperative malalignment (hip-knee-ankle angle ≥10° of varus), postoperative untargeted alignment (hip-knee-ankle angle <2° or >6° of valgus), and preoperative osteoarthritis severity (Kellgren-Lawrence grade ≥3) on survivorship of HTO was evaluated using Cox regression analysis. A failure of HTO was defined as a conversion to total knee arthroplasty (TKA).

**Results:**

A total of 410 patients (463 knees) were included, with a mean follow-up of 13.0 years (range, 5.0-18.1 years) and a mean survival time of 11.2 years (range, 1.2-18.1 years) for patients who reached the endpoint of TKA. HTO survival rates at 5, 10, and 15 years postoperatively were 91%, 78%, and 60%, respectively. Multivariate Cox regression analysis showed no significant difference in survivorship between patients with increased KJLO and those with acceptable KJLO (178 vs 285 knees; hazard ratio [HR], 0.8; 95% CI, 0.6-1.1; *P* = .148), with no significant between-group difference observed in the mean follow-up length (12.9 ± 3.0 years vs 13.1 ± 3.3 years; *P* = .105). Female sex (HR, 2.0; *P* < .001) and postoperative untargeted alignment (HR, 1.6; *P* = .003) were risk factors for a conversion to TKA.

**Conclusion:**

Increased postoperative KJLO (medial proximal tibial angle ≥95°) had no significant influence on the survivorship of lateral closing-wedge HTO. Men demonstrated superior survival outcomes compared with women, and it was important to achieve a targeted postoperative alignment (HKA 2°-6° of valgus) to ensure favorable HTO survivorship.

High tibial osteotomy (HTO) has proven to be a powerful intervention for managing patients with symptomatic medial knee osteoarthritis by realigning lower limb malalignment.^15^ Despite initial efficacy, the enduring effectiveness of HTO experiences a gradual decline over time.^1^ In cases in which HTO fails, total knee arthroplasty (TKA) presents itself as an alternative treatment option.^17^

An increased knee joint line obliquity (KJLO) has raised concerns after a valgus-producing HTO, potentially leading to biomechanical consequences of increased shear stress and an abnormal redistribution of contact stress within the knee joint.^13,23^ The medial proximal tibial angle (MPTA) has been identified as a preferable method for assessing KJLO because of its demonstrated measurement stability and reliability.^26^ An MPTA exceeding 95° postoperatively is generally considered as an excessively increased KJLO after HTO.^13^ A recent systematic review including 17 studies showed the ongoing debate revolving around the effect of postoperative increased KJLO on HTO survivorship, with limited and conflicting evidence published up to 2023,^24^ as 6 studies found an association between increased KJLO and poorer HTO survival, while 11 studies failed to confirm this association.

Pape and Rupp^16^ outlined the ideal indication for a valgus-producing HTO: patients with medial unicompartmental osteoarthritis, aged younger than 55 years, and with a preoperative varus malalignment <10°. Hui et al^8^ reported poorer HTO survival with higher TKA transition risk for patients aged older than 50 years compared with those younger than 50 years. Hence, it is intriguing to explore the potential effect of age and preoperative varus malignment on the survivorship of HTO, utilizing specified cutoff values. Moreover, as a hip-knee-ankle angle (HKA) within the range of 2°-6° of valgus has been considered as the targeted alignment for lateral closing-wedge HTO,^4,9,12^ investigating the potential effect of a correction falling outside this targeted alignment range on the survival rate of HTO holds clinical significance, addressing a notable knowledge gap. Additionally, an advanced preoperative medial knee osteoarthritis grade (Kellgren-Lawrence grade ≥3) has previously been reported to be associated with an increased risk of a conversion to TKA after HTO.^5^ Furthermore, a previous study suggested a higher risk of TKA conversion in women after HTO,^20^ and others found no sex difference,^5,6^ making the role of sex controversial in this context.

The primary purpose of this study was to investigate the influence of increased postoperative KJLO (MPTA ≥95°) on the survivorship of lateral closing-wedge HTO. The secondary purpose was to examine the effects of age, sex, perioperative HKA, and preoperative osteoarthritis severity on survivorship. We hypothesized that an excessively increased postoperative KJLO would have a negative influence on the survivorship of lateral closing-wedge HTO.

## Methods

### Study Design

This retrospective cohort study was conducted within 1 major teaching hospital located in the northern part of the Netherlands. The study design adhered to the Strengthening the Reporting of Observational Studies in Epidemiology (STROBE) guidelines.^22^ This study was approved by the local ethics committee (MEC No. 2023-105).

### Patients

Patients with symptomatic medial knee osteoarthritis and varus malalignment treated with lateral closing-wedge HTO at a single hospital were screened. The HTO was performed between January 1, 2003, and August 3, 2018. The medical records and radiographs of these patients were reviewed. Last follow-ups were conducted via outpatient clinic visits (part of usual care) or via either mail or telephone calls in 2023. All HTOs were performed by experienced knee surgeons in our orthopaedics department.

### Inclusion and Exclusion Criteria

Patients were eligible for inclusion if they met the following criteria: (1) had undergone lateral closing-wedge HTO for treating symptomatic medial knee osteoarthritis with varus malalignment; (2) had anteroposterior long-standing and short-standing radiographs, as well as lateral radiographs, taken before and after HTO; and (3) had a follow-up duration ≥5 years after HTO.

Patients were excluded if they (1) had undergone osteotomy for medial knee osteoarthritis other than lateral closing-wedge HTO, such as medial opening-wedge HTO, combined-wedge HTO, or double-level osteotomy; (2) had congenital deformity or a history of lower limb fractures or trauma surgery that could affect the radiological measurements; or (3) were deceased, refused to participate in this cohort study, or were lost to our last follow-ups because of nonresponse to mail or telephone calls.

### Surgical Technique

A lateral closing-wedge HTO was performed as described by van Raaij et al.^21^ After a transverse incision and safeguarding of the common peroneal nerve, the anterior proximal tibiofibular syndesmosis was resected, and the tibial wedge was removed using a calibrated guide. The osteotomy was stabilized with 2 staples, accompanied by a fasciotomy of the anterior compartment. The correction target was to attain an alignment of 4° of valgus after HTO.^3^

### Radiological Measurements

**Figure 1.**
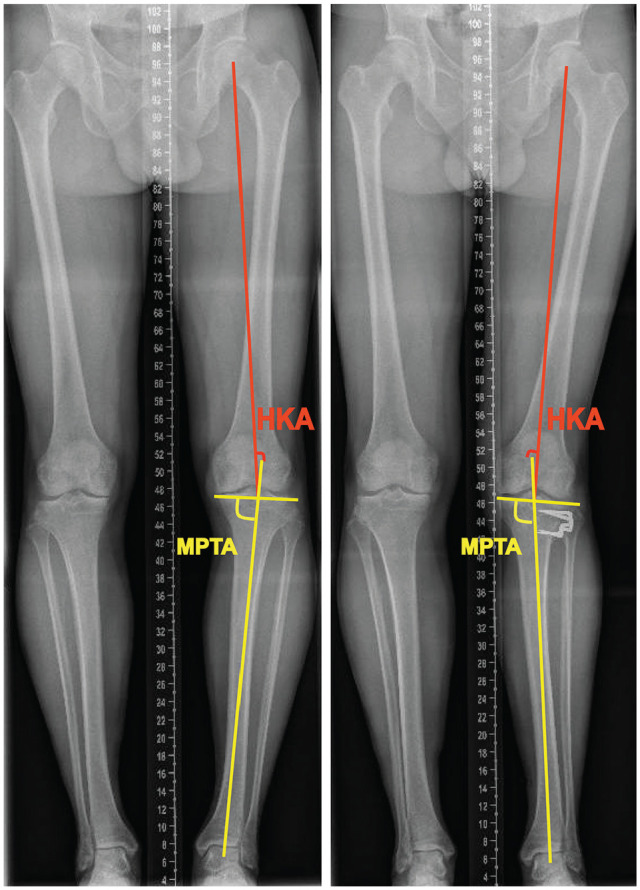
Illustration of radiological parameters: medial proximal tibial angle (MPTA) and hip-knee-ankle angle (HKA) (left panel) before and (right panel) after high tibial osteotomy.

Anteroposterior long-standing radiographs were used to measure MPTA and HKA (Figure 1), and anteroposterior short- standing knee radiographs were used to assess Kellgren-Lawrence (KL) grades. The knee was positioned in full extension with the patella facing forward during filming.

KJLO was assessed using the MPTA, which was the medial angle between the mechanical axis of the tibia and the tangential line of the tibial plateau.^26^ HKA was the angle formed by the mechanical axes of the femur and tibia.^25^ In this study, a positive value signified varus alignment, and a negative value indicated valgus alignment. An orthopaedic surgeon skilled in radiographic analysis (T.X.) conducted the MPTA and HKA measurements. In a previous study, we (T.X. and R.W.B.) found excellent intra- and interobserver measurement reliabilities (intraclass correlation coefficients >0.9) in both MPTA and HKA.^26^ KL grades were used to assess medial knee osteoarthritis severity before HTO, with 4 ordinal grades: 1 for doubtful, 2 for mild, 3 for moderate, and 4 for severe.^11^

### Grouping and Definition

Eligible patients were categorized based on different factors, including postoperative KJLO (MPTA <95° or ≥95°), age at HTO (<55 or ≥55 years), sex (male or female), preoperative malalignment (HKA <10° or ≥10° of varus), postoperative alignment (HKA 2°-6° of valgus or <2° or >6° of valgus), and preoperative osteoarthritis severity (KL grade <3 or ≥3).

An HTO failure was defined as a conversion to TKA. The HTO survival time was calculated as the duration from the date of HTO to either the date of a conversion to TKA or the last follow-up date. The postoperative complications were defined as adverse events or unintended outcomes associated with HTO.

### Statistical Analysis

SPSS software (Version 25; IBM Corp) was used for statistical analysis. Distribution of continuous data was checked using the Shapiro-Wilk test and Q-Q plot. Univariate and multivariate Cox regression analyses were used to generate hazard ratios (HRs) and their corresponding 95% confidence intervals for potential risk factors, including postoperative KJLO (MPTA <95° or ≥95°), age (<55 or ≥55 years), sex (male or female), preoperative alignment (HKA <10° or ≥10° of varus), postoperative alignment (HKA 2°-6° of valgus or <2° or >6° of valgus), and preoperative osteoarthritis severity (KL grade <3 or ≥3). The Kaplan-Meier analysis was used for determining the survival rate at 5, 10, and 15 years postoperatively. Continuous data are reported as mean ± standard deviation, and categorical data are presented as number and frequency. A *P* value of <.05 signified statistical significance. A post hoc power analysis was conducted, showing that with 178 patients in the increased KJLO group (MPTA ≥95°) and 285 patients in the acceptable KJLO group (MPTA <95°), the study would possess a statistical power of 84.5% at an alpha level of .05, enabling the detection of a 10% difference in HTO survival between the 2 groups.

## Results

**Figure 2.**
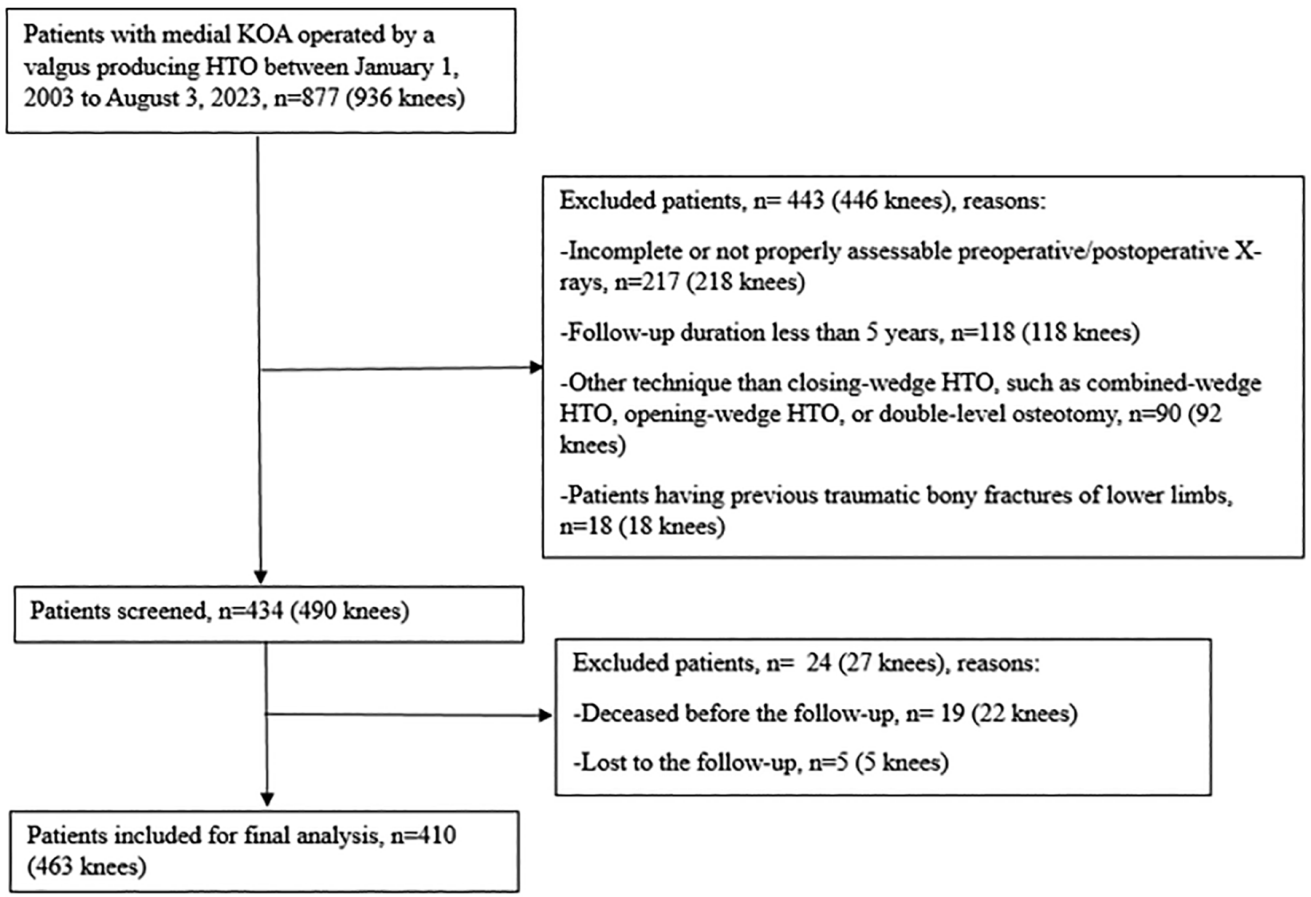
Flowchart of patient selection process. HTO, high tibial osteotomy; KOA, knee osteoarthritis.

The patient selection process is illustrated in Figure 2. After applying the predefined inclusion and exclusion criteria, we included a total of 410 patients with 463 knees. Patient baseline characteristics are described in Table 1. The mean patient age at the time of HTO was 52.1 ± 7.5 years. At the last follow-up, 159 knees (34%) had received a conversion to TKA. The mean HTO survival time was 11.2 ± 4.1 years (range, 1.2-18.1 years) for patients who had reached the endpoint of TKA. The mean follow-up length after HTO was 13.0 ± 3.2 years (range, 5.0-18.1 years). There was no significant difference observed in the mean follow-up length after HTO between the MPTA <95° group (285 knees; 13.1 ± 3.3 years) and the MPTA ≥95° group (178 knees; 12.9 ± 3.0 years) (*P* = .105).

**Table 1:** Patient Characteristics Before and After HTO^*a*^

	Value
No. of included knees	463
Age at HTO, y	52.1 ± 7.5
Sex, male/female, n (%)	298 (64)/165 (36)
Operated side, left/right, n (%)	238 (51)/225 (49)
Preoperative HKA, deg	5.1 ± 2.5
Postoperative HKA, deg	−2.4 ± 3.5
Preoperative MPTA, deg	86.9 ± 2.2
Postoperative MPTA, deg	94.0 ± 3.3
Preoperative KL grades, 1/2/3/4, n (%)	18/382/58/5 (4/82/13/1)

a Data are shown as mean ± SD unless indicated otherwise. HKA, hip-knee-ankle angle; HTO, high tibial osteotomy; KL, Kellgren-Lawrence; MPTA, medial proximal tibial angle.

**Figure 3.**
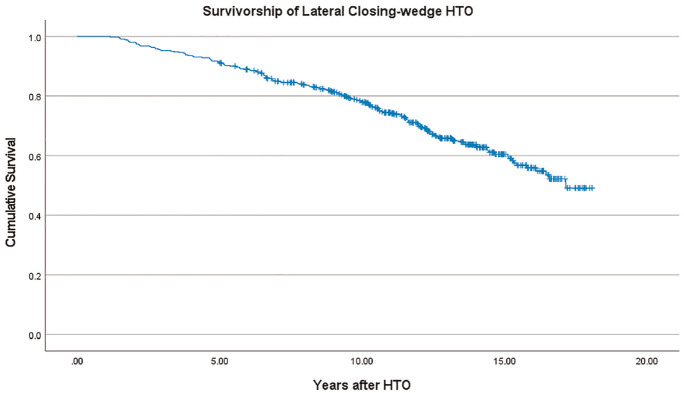
Kaplan-Meier survival analysis for 463 knees after lateral closing-wedge high tibial osteotomy (HTO).

The HTO survivorship assessed using Kaplan-Meier analysis is shown in Figure 3, with survival rates of 91.1%, 77.9%, and 59.7% at 5, 10, and 15 years, respectively. The complication rate after HTO was 3.3%, as presented in Table 2. The influences of potential risk factors on HTO survivorship analyzed using univariate and multivariate Cox regression models are shown in Table 3.

**Table 2:** Complications After High Tibial Osteotomy^*a*^

	No. of Cases (%)
Resurgery for recurrent varus malalignment	9 (1.9)
Nonunion	2 (0.4)
Delayed union	2 (0.4)
Deep venous thrombosis	2 (0.4)
Compartment syndrome	1 (0.2)
Total	16 (3.3)

a A resurgery denotes either a reoperated high tibial osteotomy or conversion to total knee arthroplasty.

**Table 3:** Influences of Factors on a Conversion to TKA After HTO Using Univariate and Multivariate Cox Regression Analyses^*a*^

		Survival, %			HR (95% CI)^*b*^		*P* Value	
	No. of Knees	5 y	10 y	15 y	Univariate Regression	Multivariate Regression	Univariate Regression	Multivariate Regression
Postoperative MPTA								
<95°	285	90.2	76.9	56.2	0.8 (0.5-1.1)	0.8 (0.6-1.1)	.111	.148
≥95°	178	92.7	79.6	64.2				
Age at HTO								
<55 y	268	92.2	80.9	61.1	1.2 (0.9-1.6)	1.3 (0.9-1.7)	.250	.158
≥55 y	195	89.7	73.9	55.7				
Sex								
Male	298	94.6	82.7	65.9	1.9 (1.4-2.6)	2.0 (1.5-2.8)	**<.001**	**<.001**
Female	165	84.8	69.6	46.8				
Preoperative HKA								
<10° of varus	449	91.1	78.2	60.8	1.9 (0.9-3.8)	2.0 (1.0-4.1)	.087	.065
≥10° of varus	14	85.7	70.7	34.4				
Postoperative HKA								
2°-6° of valgus	229	95.6	83.1	68.3	1.7 (1.3-2.4)	1.6 (1.2-2.3)	**<.001**	**.003**
<2° or >6° of valgus	234	86.3	72.9	51.3				
Preoperative KL grade								
<3	400	92.0	78.9	61.2	1.5 (1.0-2.3)	1.4 (0.9-2.2)	.059	.099
≥3	63	85.7	69.9	48.1				

a Bold *P* values indicate statistical significance. HKA, hip-knee-ankle angle; HR, hazard ratio; HTO, high tibial osteotomy; KL, Kellgren-Lawrence; MPTA, medial proximal tibial angle; TKA, total knee arthroplasty.

b The hazard ratio is calculated relative to the first category for each variable.

In both univariate and multivariate Cox regression analyses, postoperative increased KJLO (MPTA ≥95°) was not associated with a conversion to TKA after HTO (HR, 0.8 and 0.8; respectively; *P* > .05), whereas female sex (HR, 1.9 and 2.0, respectively; *P* < .001) and postoperative untargeted alignment (HKA <2° or >6° of valgus) (HR, 1.7 and 1.6, respectively; *P* < .01) were significant risk factors for a conversion to TKA after HTO.

In both univariate and multivariate Cox regression analyses, age at HTO (≥55 years), preoperative malalignment (HKA ≥10° of varus), and preoperative medial knee osteoarthritis severity (KL grade ≥3) were not associated with a conversion to TKA after HTO (*P* > .05).

## Discussion

The most important finding of this study was that an excessively increased postoperative KJLO (MPTA ≥95°) did not influence the survivorship of lateral closing-wedge HTO. Female sex and postoperative untargeted alignment (HKA <2° or >6° of valgus) were significant risk factors associated with a conversion to TKA after HTO. The present finding rejects our hypothesis that an excessively increased KJLO would have a negative influence on survivorship of lateral closing-wedge HTO.

Although an excessively increased KJLO after HTO has been related to abnormal biomechanical effects such as shear stress increase and contact loading redistribution,^13,23^ in this study, increased KJLO did not affect the survivorship of a lateral closing-wedge HTO. Babis et al^2^ found that a postoperative KJLO (measured using the joint line orientation angle) <4° was associated with an improved 10-year survival rate of lateral closing-wedge HTO in 10 knees. However, this joint line orientation angle is influenced by the filming technique used (single-leg/double-leg standing, feet distance), which has been proven to not be an ideal method for assessing KJLO.^26^ Schuster et al^19^ found no significant difference in the 10-year survival rate of medial opening-wedge HTO when comparing postoperative MPTA ≤95° and >95° (64 vs 15 patients). Also, no relationship between KJLO and clinical (patient-reported) outcomes was found. Rosso et al^18^ evaluated this relationship in 92 knees with opening-wedge HTO at 10 years of follow-up and found that an increased KJLO (MPTA ≥95°) was not correlated with clinical outcomes like the Western Ontario and McMaster Universities Osteoarthritis Index and Knee Society Score. Xie et al^25^ found no significant difference in 5-year survival rate of lateral closing-wedge HTO when comparing postoperative MPTA <95° with ≥95° (58 patients in each group). However, the above studies were constrained by their inclusion of a limited number of patients and the absence of a regression model for survival analysis. Furthermore, it is important to recognize biomechanical differences between closing-wedge and opening-wedge HTOs,^14^ as the former create lateral defect laxity that may affect HTO survival.^25^ Notably, although previous KJLO studies have mostly focused on medial opening-wedge HTO,^24^ our study highlighted no influence of increased KJLO on survival after lateral closing-wedge HTO.

The present study demonstrates that female sex and an untargeted postoperative alignment (HKA <2° or >6° of valgus) are risk factors associated with deteriorated survivorship of lateral closing-wedge HTO. Our finding aligns with the discovery of van Raaij et al^20^ indicating that female patients undergoing lateral closing-wedge HTO exhibit inferior survival rates compared with their male counterparts (100 cases; mean follow-up, 12 years). The observed between-sex difference may be attributed to hormonal variations, with higher testosterone levels in men potentially enhancing muscle strength and overall joint stability, resulting in improved post-HTO survival. However, Efe et al^5^ (199 cases; mean follow-up, 10 years) and Gstöttner et al^6^ (134 cases; mean follow-up, 12 years) did not identify a survival difference in HTO between men and women (*P* > .05). Jin et al^10^ identified that a postoperative HKA <0°, indicating an undercorrection, was associated with HTO failure necessitating conversion to TKA. Both our study and that of Jin et al^10^ underscore the importance of achieving a targeted postoperative valgus alignment in HTO.

Our study found that age ≥55 years, preoperative HKA ≥10° of varus, and preoperative KL grade ≥3 were not significantly associated with HTO survival. This finding aligns with the conclusion of Gstöttner et al^6^ suggesting that the preoperative HKA varus degree is not associated with survival of lateral closing-wedge HTO. This implies that HTO prioritizes correcting varus alignment for a specific postoperative alignment, emphasizing the critical role of successful correction over the initial varus degree. However, it is important to note that our study had an imbalance in patient numbers between groups, with 449 patients in the preoperative HKA <10° group and only 14 patients in the ≥10° group. This imbalance can compromise the statistical power during the comparison, thereby impeding the ability to draw strong conclusions from it. Contrary to the present finding, Efe et al^5^ identified high preoperative osteoarthritis severity (KL grade ≥3) as a risk factor for lateral closing-wedge HTO failure (199 cases; mean follow-up, 10 years). There are differences in KL grade distribution in the present study (1/2/3/4, 4%/82%/13%/1%) and the study by Efe et al^5^ (1/2/3, 49%/48%/3%). In the study by Efe et al,^5^ half of the patients had doubtful osteoarthritis (KL grade 1) and none had severe osteoarthritis. The differences in KL grade distribution may explain variations in the relationship between KL grade and HTO survivorship in our study versus that by Efe et al.^5^ Furthermore, our findings do not align with the recommended indications proposed by Pape and Rupp,^16^ as we did not observe improved long-term survivorship with age younger than 55 years and preoperative varus malalignment <10°. The age may not be a stringent criterion for defining the ideal indications in HTO. Future HTOs might adopt a more inclusive set of indications.

Lateral closing-wedge HTO demonstrated favorable survivorship outcomes. Efe et al^5^ reported survival rates of 93%, 84%, and 68% at 5, 10, and 15 years, respectively, and Gstöttner et al^6^ found survival rates of 94%, 80%, and 66% at 5, 10, and 15 years, respectively. The present study showed survival rates marginally lower than those reported in the aforementioned studies, with rates of 91%, 78%, and 60% at 5, 10, and 15 years, respectively. These results highlight lateral closing-wedge HTO as a potent and enduring surgical technique for medial knee osteoarthritis with varus malalignment, maintaining a sustained effectiveness at 10 years in around 80% of patients who received operation.

A surgical intervention (second HTO or conversion to TKA) for recurrence of varus malalignment within the 5 years after lateral closing-wedge HTO was the most common complication (1.9%) in the present study. In a study by Hui et al,^8^ deep venous thromboses (2%) and pulmonary emboli (1%) were reported as the most common complications in 394 cases after a lateral closing-wedge HTO. In a study by Howells et al,^7^ delayed union (3%), superficial wound infection (2%), common peroneal nerve palsy (1%), and pulmonary emboli (1%) were complications in 95 cases after a lateral closing-wedge HTO. The present study observed a deep venous thrombosis rate of 0.4%, signifying a lower incidence within the study cohort than found in Hui et al^8^ and Howells et al.^7^ Additionally, zero cases of peroneal nerve palsy were observed in our included patients. This was achieved by carefully exposing and protecting the peroneal nerve while leaving the fascia of this compartment open. Moreover, although our correction aimed for an alignment of 4° of valgus, there were instances in which undercorrection was noted. The reccurrence of varus malalignment may be attributed to this undercorrection.

To the best of our knowledge, this cohort study stands out as the first in which the Cox regression model was used in assessing the influence of KJLO on HTO survival rates. It boasts the largest patient sample among current published studies, encompassing 463 HTO-operated knees. Additionally, this study benefits from a significant follow-up duration, with a mean of 13 years (range, 5-18 years).

This study has limitations. The body mass index data for our included patients were incomplete, precluding a comprehensive analysis. The potential influence of body mass index on the survival of HTO remains a significant consideration.^7,8^ Furthermore, we lacked complete patient-reported outcome data before and after HTO, so our focus was on the association between KJLO and HTO survivorship, not on patient-reported outcomes.

## Conclusion

Increased postoperative KJLO (MPTA ≥95°) had no significant influence on the survivorship of lateral closing-wedge HTO. Men demonstrated superior survival outcomes compared with women, and it was important to achieve a targeted postoperative alignment (HKA 2°-6° of valgus) to ensure favorable HTO survivorship.
